# Iron Oxide Nanoparticles: Parameters for Optimized Photoconversion Efficiency in Synergistic Cancer Treatment

**DOI:** 10.3390/jfb15080207

**Published:** 2024-07-25

**Authors:** Tsenka Grancharova, Plamen Zagorchev, Bissera Pilicheva

**Affiliations:** 1 Department of Medical Physics and Biophysics, Faculty of Pharmacy, Medical University of Plovdiv, 4002 Plovdiv, Bulgaria; tsenka.grancharova@mu-plovdiv.bg (T.G.); plamen.zagorchev@mu-plovdiv.bg (P.Z.); 2 Research Institute, Medical University of Plovdiv, 4002 Plovdiv, Bulgaria; 3 Department of Pharmaceutical Sciences, Faculty of Pharmacy, Medical University of Plovdiv, 4002 Plovdiv, Bulgaria

**Keywords:** iron oxide, photothermia, theranostic, synergistic, cancer, nanomedicine, nanoparticles

## Abstract

Photothermal therapy (PTT) can overcome cancer treatment resistance by enhancing the cell membrane permeability, facilitating drug accumulation, and promoting drug release within the tumor tissue. Iron oxide nanoparticles (IONPs) have emerged as effective agents for PTT due to their unique properties and biocompatibility. Approved for the treatment of anemia, as MRI contrast agents, and as magnetic hyperthermia mediators, IONPs also offer excellent light-to-heat conversion and can be manipulated using external magnetic fields for targeted accumulation in specific tissue. Optimizing parameters such as the laser wavelength, power density, shape, size, iron oxidation state, functionalization, and concentration is crucial for IONPs’ effectiveness. In addition to PTT, IONPs enhance other cancer treatment modalities. They improve tumor oxygenation, enhancing the efficacy of radiotherapy and photodynamic therapy. IONPs can also trigger ferroptosis, a programmed cell death pathway mediated by iron-dependent lipid peroxidation. Their magneto-mechanical effect allows them to exert a mechanical force on cancer cells to destroy tumors, minimizing the damage to healthy tissue. This review outlines strategies for the management of the photothermal performance and PTT efficiency with iron oxide nanoparticles, as well as synergies with other cancer therapies.

## 1. Introduction

Due to its complexity, heterogeneity, and ability to migrate and invade distant sites, cancer remains one of the major challenges in medicine today. The current conventional treatments for tumors include chemotherapy (CT), surgery, and radiotherapy (RT), and, despite their reported success, they are accompanied by inevitable side effects. These treatments do not always achieve the desired outcome of preventing tumor recurrence and metastasis and, in some cases, may exhibit prometastatic effects [1,2]. Furthermore, cancer cells are capable of metabolic changes that lead to resistance to RT and CT [3]. As a result, cancer remains the second leading cause of death worldwide.

Nanotechnology offers a promising way to address the aforementioned challenges in cancer therapy. Nanoparticles exhibit different properties compared to their respective bulk materials, which can facilitate interactions with cell surfaces and intercellular structures [4]. Nanoparticles (NPs) can be engineered as therapeutic agents with versatile properties: enhancing biodistribution, preventing the encapsulated cargo from degradation, incorporating targeting ligands, and overcoming drug resistance [5]. The appropriate design also leads to the theranostic potential of NPs and enables multimodal therapy. Such integration of therapies results in synergistic action, leading to improved patient outcomes.

**Figure 1:**
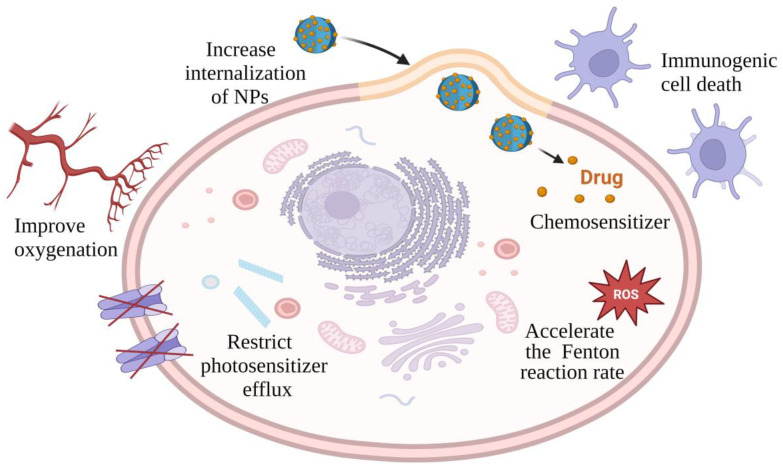
The role of hyperthermia in cancer therapy. Created with Biorender.com.

An example of a candidate for synergistic therapy is hyperthermia (Figure 1), as it has demonstrated the potential to enhance the effects of RT and CT [6]. Hyperthermia also triggers the release of heat shock proteins, activating immune cells [7] and further improving the therapeutic outcome. Furthermore, an elevated temperature itself serves as an effective therapy by inducing protein denaturation, causing indirect DNA damage [8]. Hyperthermia also induces reactive oxygen species (ROS) production in tumor cells, cell cycle arrest, cytoskeletal alterations and damage to collagen fibers, changes in the expression of tumor cell genes, and microvessel damage [9]. Owing to rapid angiogenesis, the blood vessels in tumor tissue exhibit abnormal morphological growth, compromised functionality, and challenges in dissipating accumulated heat. As a result, tumor tissue exhibits higher sensitivity to hyperthermia compared to healthy tissue. Researches have demonstrated that the exposure of cancer cells at 43 °C for 30–60 min can result in effective destruction, with this duration being shortened with an increase in the temperature.

The integration of nanotechnology into the field of hyperthermia has led to the development of innovative approaches such as magnetic hyperthermia (MH) and photothermia. Photothermal therapy (PTT) relies on the absorption of electromagnetic waves by photothermal agents and their conversion into heat. Various materials can absorb electromagnetic radiation, causing electron excitation from their ground to their first singlet excited state. These excited electrons can then relax either radiatively, emitting light as fluorescence or phosphorescence, or nonradiatively, by converting energy into heat (photothermal conversion) through certain paths [10]. The absorption of light in metals leads to the coherent oscillation of free electrons at the surface (surface plasmon resonance, SPR). The energy from these oscillations raises the temperature of the material. In semiconductors, electromagnetic absorption generates electron–hole pairs and their recombination leads to heat production. Moreover, π-conjugated materials elevate the temperature through molecular vibrations.

Two main parameters describe the photoconversion ability of materials. The specific absorption rate (SAR) measures the rate at which energy is absorbed by a unit mass of material, expressed in watts per gram (W/g).

(1) $$\mathrm{SAR}=C\frac{\Delta T}{\Delta t}\frac{m_{s}}{m_{NPs}}$$

For the laser irradiation of aqueous dispersions of NPs, *C* is the specific heat capacity of water; Δ*T*/Δ*t* is the initial slope of the temperature curve as a function of time; and *m_s_* and *m_NPs_* are the masses of the solvent and the NPs, respectively.

The other parameter, the photothermal conversion efficiency η, determines the effectiveness of photoconversion.

(2) $$\eta =\frac{hs\left(T_{max}-{\;T}_{surr}\right)-{\;Q}_{diss}}{I\left(1-10^{A\lambda }\right)}$$

where *h* is the heat transfer coefficient, *s* is the surface area of the container, *T_ma_*_x_ − *T_surr_* is the temperature change of the NP dispersion during irradiation, *Q_diss_* is the heat dissipated from the light absorbed by the quartz sample cell itself, *I* is the laser power density, and *Aλ* is the absorbance at the laser wavelength.

Utilizing NPs with high conversion efficiency generates nanoscale hotspots, elevating the temperature and focusing the effect of hyperthermia on the tissue where they accumulate. Among the photothermal agents, gold NPs represent the gold standard for PTT because of their high light-to-heat conversion efficiency. Gold-shelled silica-core NPs, known as AuroShell^®^, have reached clinical trials for the treatment of metastatic lung cancer (NCT01679470), head and neck cancer (NCT00848042), and prostate cancer [11] (NCT02680535 and NCT04240639). PTT using gold-silica NPs is also being investigated for the treatment of atherosclerosis (NCT01270139) and acne (NCT02219074). Iron oxide nanoparticles (IONPs) are also desirable photothermal agents due to their unique properties and excellent biocompatibility. They have already been approved for the treatment of anemia, as an MRI contrast agent, and as a mediator for MH. Studies have shown that IONPs possess good light-to-heat conversion abilities. An additional advantage is that the manipulation of IONPs using an external magnetic field could facilitate targeted accumulation in particular tissue types, contribute to their theranostic potential, and offer an opportunity for multimodal therapy.

The aim of the present review is to summarize the recent findings on the biomedical applications of IONPs and outline the challenges and future perspectives in the field.

## 2. Iron Oxides in Medicine

**Figure 2:**
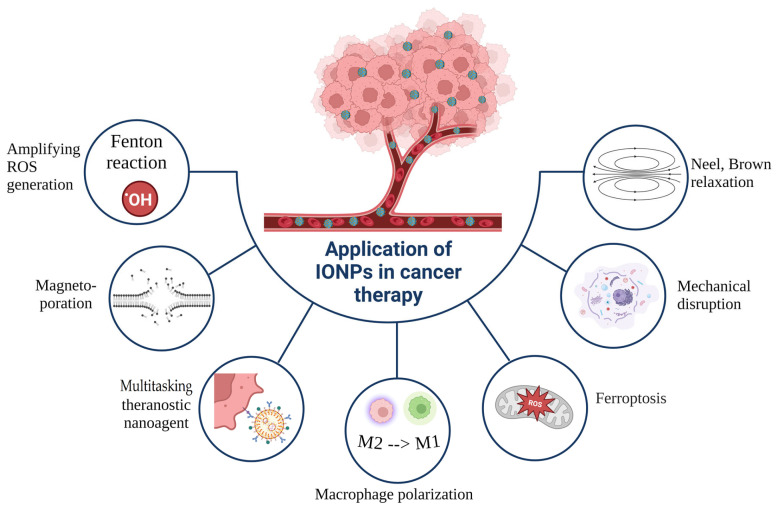
Application of IONPs in cancer therapy. Created with Biorender.com.

Iron oxides and oxyhydroxides exist in various forms, and magnetite (Fe_3_O_4_) and maghemite (γFe_2_O_3_) are the most frequently investigated for biomedical applications. The medical uses of IONPs include anemia treatment, magnetic resonance imaging (MRI), cell separation, MH, and drug delivery. The inherent superparamagnetism of IONPs enables their manipulation using external magnetic fields. IONPs display excellent biodegradability, as their iron content can be assimilated by the body for future physiological needs. An interesting characteristic is their “nanozyme” activity, as they possess intrinsic peroxidase-like behavior [12]. These properties allow IONPs to serve as a versatile platform, capable of addressing diverse medical needs (Figure 2). For example, ferumoxytol, an IONP formulation, is approved for anemia treatment and also as an MRI contrast agent with T1, T2, and T2* shortening capabilities. Beyond these, ferumoxytol demonstrates great potential in numerous other medical applications [13], including drug delivery, oral biofilm treatment, and anti-cancer and anti-inflammatory therapies. In the presence of hydrogen peroxide (H_2_O_2_), ferumoxytol generates ROS, which have the potential to trigger ferroptosis—a type of iron-dependent cell death—and has been explored by some researchers as a cancer therapy [14].

## 3. Synthesis of IONPs

IONPs can be synthesized using a variety of physical, chemical, and biological methods (Table 1). The choice of the synthesis technique is important, as it affects the parameters and properties of the NPs, as well as meeting the specific requirements of different applications. Each method has its advantages and is suitable for the production of IONPs with specific characteristics. Physical methods for the synthesis of IONPs involve the use of thermal energy, mechanical pressure, etc., to induce material condensation, evaporation, abrasion, or melting [15]. Compared to chemical approaches, physical methods offer several advantages, including the absence of contamination and the homogeneous distribution of the NPs. However, they often require specialized equipment. Common physical techniques for the generation of IONPs include laser ablation or pyrolysis, physical vapor deposition, high-energy ball milling, etc. The chemical synthesis of NPs uses inorganic and organic agents to reduce ions and form oligomeric clusters. To stabilize these NPs and prevent agglomeration, protective agents are essential. The various chemical methods for nanoparticle creation include coprecipitation, hydrothermal synthesis, solvothermal synthesis, polyol synthesis, microemulsion techniques, sol–gel methods, and plasma and chemical vapor synthesis.

In their study, Luo et al. [16] used the laser ablation of iron in a vortex fluidic device for the synthesis of hexagonal and spheroidal SPIONs with an average size of 15 nm. Feng et al. [17] developed 14 nm porous hollow copper IONPs with 2–3 nm pores for use as a cisplatin carrier and PTT agent. These nanoagents were prepared via a galvanic reaction between Cu_2_S nanoparticles and iron pentacarbonyl, followed by etching to produce the pores. The controlled release of Fe and Cu ions enhanced chemodynamic therapy. Wang et al. [18] created multifunctional carbon-encapsulated Au–Fe_3_O_4_ nanoaggregates using enclosed flame spray pyrolysis, which exhibited excellent MRI capabilities and photothermal performance. Byeon et al. [19] fabricated Au-decorated IONPs via a single-pass aerosol route for CT and MR imaging, gene delivery, and PTT. Ji et al. [20] developed an IONP-loaded alginate hydrogel for the PTT of colorectal cancer cells, with Fe_3_O_4_ NPs synthesized via coprecipitation. Liu et al. [21] demonstrated the photothermal effect of Ag@Fe_3_O_4_ synthesized via the hydrothermal method. Shaw et al. [22] used a microwave-assisted polyol method to fabricate γ-Fe_2_O_3_ nanoflowers. Varying amounts of sodium acetate as an alkali source allowed the modification of the magnetic and structural properties, resulting in nanoflowers with high heating performance for MH and PTT.

IONPs can also be produced by using bacteria, fungi, and plant extracts. Green synthesis using plant polyphenols was reported for the fabrication of a hollow CoPt MRI and PA imaging-guided PTT nanoagent [24]. This strategy enables size-controlled NP synthesis through the control of the molecular sizes of polyphenols and is versatile for the synthesis of other alloy nanoparticles due to the metal-chelating ability of the polyphenols. Iron–gold Auroshell nanoparticles were synthesized using a rosemary extract, designed for hyperthermia applications using a hot water bath [25]. Kharey et al. [23] reported the cost-effective green synthesis of multifunctional and biocompatible Au–IONPs using Pimenta dioica. These nanoparticles were successfully applied as contrast agents for MRI and as therapeutic agents for MH and PTT.

Cell membranes have been used to create biomimetic nanoparticles for a “Trojan horse” strategy, providing effective camouflage, enabling immune escape, prolonging the circulation time, and enhancing targeted drug delivery. By using their natural interactions with in vivo environments, erythrocytes, leukocytes, mesenchymal stem cells, and platelets have been utilized to coat nanoagents for various purposes in cancer treatment. More information about the application of cell membrane-camouflaged NPs for PTT can be found in [26,27].

## 4. Parameters That Affect IONP-Mediated PTT

**Figure 3:**
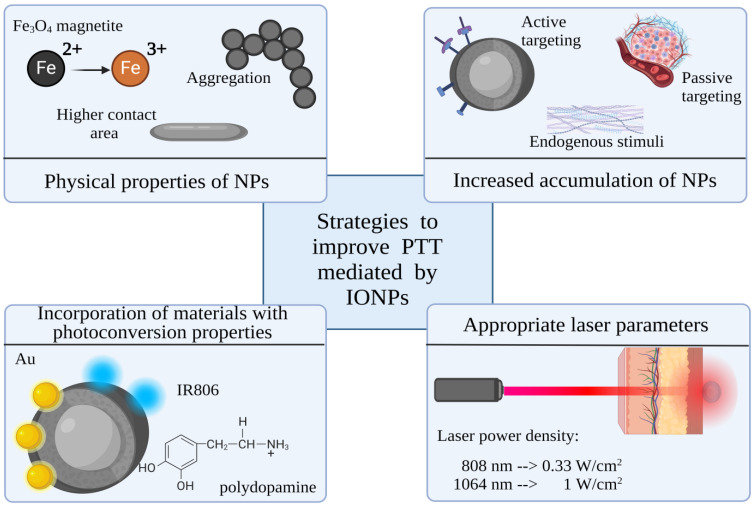
Parameters that affect the photothermal performance of IONPs. Created with Biorender.com.

Due to their good photothermal conversion capabilities and the aforementioned characteristics, IONPs have garnered increased attention as a mediator for photothermal therapy, with theranostic potential and suitability for combined therapies. The proper selection of the IONPs’ properties and functionalization plays an important role in achieving a photothermal effect (Figure 3). This significance is amplified when the IONP must exhibit multiple abilities. The selection of optimized parameters for multifunctional IONPs should be based on the required effectiveness for each function.

### 4.1. Laser Irradiation

Parameters such as the power density and laser wavelength are essential for the efficacy of PTT. Near-infrared (NIR) light is predominantly used because of the lower tissue absorption and scattering [28]. Radiation within the range of 650–900 nm, referred to as the first biological NIR window, and that between 1000 nm and 1350 nm, known as the second window, enables deep tissue penetration. While the focus has predominantly been on NIR-I, the NIR-II window exhibits potential for greater tissue penetration. Wu et al. demonstrated the greater transmittance of 1275 nm over 808 nm laser irradiation, due to the reduced tissue scattering and limited absorption by melanin, hemoglobin, and other human tissue [29]. Furthermore, NIR-II possesses higher maximum permissible exposure compared to NIR-I. Generally, for safe skin exposure during continuous laser irradiation, the clinical limits are considered to be a laser power density below 0.33 W/cm^2^ for 808 nm lasers and 1 W/cm^2^ for 1064 nm lasers [30]. Increased power of laser irradiation is directly correlated to elevated temperatures in photothermia.

While the biological windows offer enhanced tissue permeability, only a couple of millimeters of tissue are conducive to PTT. Hirsch reported treatment depths of 4 to 6 mm [31], proposing that the depth could be further extended by combining low concentrations of NPs with prolonged irradiation periods. The penetration depth of the laser into the tissue is restricted at high concentrations due to light absorption by the NPs. As a result, lower NP concentrations facilitate deeper NIR penetration into the tumor. Additionally, the maximum treatable depth is influenced by tissue perfusion [32] and pigmentation. In cases where tumors are not easily accessible and their depth exceeds 1 cm, techniques such as endoscopy or interstitial fiber-optic lasers are employed to deliver NIR to the tumor site.

### 4.2. IONPs’ Shape and Size

In contrast to plasmonic NPs, for semiconductors such as IONPs, the photothermal conversion mechanism is primarily due to electronic transitions between the d orbitals of neighboring Fe ions [33,34] and seems to be independent of the particle size and shape [35,36]. However, the size and the shape can affect the interactions of NPs with infrared laser radiation. A higher surface-to-volume ratio and larger contact area correspond to an increased probability of interaction of NIR photons with NPs [37]. Guo et al. [35] investigated the photothermal capabilities of spherical IONPs ranging in size from 60 to 310 nm. They observed that the size of these NPs did not significantly impact the temperature increase in suspensions. However, the in vivo study revealed that the 310 nm NPs showed the greatest increase in temperature, attributed to their good tumor retention. In another study, Espinosa et al. [34] explored the photothermal and therapeutic properties of various NPs, including rock-like maghemite NPs (9–11 nm), magnetite nanocubes (20 nm), maghemite nanoflowers (25 nm), cobalt ferrite NPs, and plasmonic NPs. The results showed that, among the IONPs, the cube-shaped ones exhibited the most substantial increase in temperature. Peng et al. [38] investigated the photothermal potential of Fe_3_O_4_ particles stabilized with various ligands. The experiment revealed that as the particle size increased from 120 to 380 nm, the temperature change also increased. This temperature change was attributed to the redshift in their Vis–NIR spectra, leading to an enhanced photothermal effect. Freis et al. [33] studied the effect of the shapes of IONPs (12-nm-sized nanospheres, 14- and 18-nm-sized nanocubes, and 30-nm-long nanoplatelets) on their performance as PTT agents. Their findings indicated that all NPs heated up intensively when exposed to NIR light. However, the study did not determine the specific effects of the shape and size on the heating ability.

Additionally, research has indicated that an increase in size due to aggregation is directly related to the enhanced photothermal capabilities of the IONPs, which is of great significance for the cellular uptake of NPs through endosomes. While aggregation results in reduced heating capacity in the context of MH [39], optical hyperthermia benefits from increased aggregation [40].

### 4.3. Form of Iron Oxide

Magnetite and maghemite IONPs exhibit different absorption profiles, especially in the NIR-II region, where an absorption band is evidenced only for magnetite. For maghemite, characterized by oxidized Fe cations (Fe^3+^), the optical response in the NIR region is reduced because of the absence of charge transfer transitions between the Fe^2+^ and Fe^3+^ ions in the crystal lattice. The abundance of magnetite correlates to higher optical absorption compared to maghemite [34,36,41]. In their study, Peng et al. [38] demonstrated that Fe_3_O_4_ particles stabilized by macromolecular ligands outperformed those stabilized by small molecular ligands in terms of photothermal effects. This was attributed to their ability to prevent the oxidation of magnetite to maghemite. It was also highlighted that prolonged storage can lead to the conversion of magnetite to maghemite, resulting in a significant reduction in photothermal performance. Additionally, Cabana et al. [41] indicated that magnetite is more effective as a heater in photothermal applications compared to maghemite, and they demonstrated its potential for combined MH and PTT. In another study, Gulzar et al. [42] investigated the photothermal performance of hematite (α-Fe_2_O_3_) nanorods in vitro and in vivo. They reported the good photothermal capacity of the NPs, leading to an effective reduction in the tumor size.

### 4.4. Concentration of IONPs

It is widely reported that an increase in the concentration of IONPs corresponds to an elevation in the temperature change in the sample. In contrast to the temperature, the specific absorption rate (SAR) appears to exhibit a decrease with increasing concentrations [33]. The concentrations of IONPs employed in research studies vary widely, ranging from µg/mL to mg/mL, depending on the specific application. Thus, a consensus regarding the most suitable concentrations to be used has not been reached. In their detailed review, Southern and Pankhurst [43] summarized the dosage limits for both preclinical and clinical studies involving magnetic NPs used for MRI and MH. For intravenous administration, they found that the total daily Fe dosage might reach up to 2.5 mg Fe per kg of body weight. In the case of interstitial administration, the local Fe dose can reach up to 12 mg Fe/mL tissue per site for subcutaneous injections and up to 40 mg Fe/mL tissue per site for intratumoral injections.

### 4.5. Accumulation of IONPs

The effectiveness of the therapy depends on the ability of the NPs to accumulate in the target tissue. NPs are internalized into tissue by active or passive mechanisms. The passive mechanism involves the accumulation of NPs due to the morphological and physiological differences between tumor and normal tissue, such as the enhanced permeability and retention (EPR) effect [4]. The EPR occurs due to the lack of functional lymphatic vessels, which allows NPs to remain in the tumor tissue for an extended period of time. Additionally, the wider pores between the endothelial cells in tumors compared to those of normal blood vessels contribute to the EPR. While the pore sizes of healthy blood vessels are below 6 nm, tumor vessels exhibit pore sizes of about 780 nm [44]. To achieve prolonged circulation and a higher probability of accumulation in the target tissue, the size of the NPs should be between 15 nm and 100 nm [45]. NPs smaller than 100 nm avoid accumulation in the liver and the spleen, while NPs larger than 10 nm are less susceptible to renal clearance. The selection of the appropriate size is also based on the diffusion through the tumor tissue. NPs above 50 nm have difficulties in migrating beyond the perivascular regions of the tumor [46] and tend to accumulate around tumor blood vessels.

An alternative strategy to enhance accumulation involves cell membrane coating, where naturally derived cell membranes are utilized to encapsulate NPs [47]. This technique imparts biomimetic functions to the NPs that replicate the biological characteristics inherited from their source cells. For example, it seamlessly integrates the immune evasion capabilities of platelets and erythrocytes, as well as the tumor-targeting abilities of immune cells, stem cells, and cancer cell membranes [48]. Moreover, biomimetic NPs offer significant advantages, including minimal cytotoxicity, enhanced biocompatibility, and structural reinforcement. Ren et al. [49] reported the preparation of a red blood cell (RBC)-derived membrane coating for their imaging-guided IONP photothermal platform. The RBC membrane-camouflaged NPs showed prolonged blood circulation and good properties in vivo.

In certain cases, especially during the early stages, some tumors might not exhibit the EPR effect. To address this, active targeting mechanisms have been developed to achieve more precise targeting. Active targeting involves attaching ligands such as antibodies, peptides, nucleic acids, proteins, vitamins, and carbohydrates to the surfaces of the NPs. These ligands then bind to the receptors that are overexpressed by the tumor cells. The constant need for substances to support the rapid proliferation of cancer cells leads to the overexpression of specific receptors, such as transferrin, folate receptors, epidermal growth factor receptors (EGFRs), cluster of differentiation (CD) receptors, and more [50]. Larson et al. [51] designed gold-coated IONPs for the imaging and PTT of cancer cells, exhibiting a specific affinity for EGFR—a biomarker linked to numerous epithelial cancers. In another study, Lu et al. [52] developed Fe_3_O_4_–graphene oxide NPs with dual targeting capabilities for CT–PTT. These NPs were modified with cetuximab, an EGFR monoclonal antibody. Yand et al. [53] also investigated NPs with dual-modal imaging and photothermal conversion abilities with hyaluronic acid functionalization, with effectiveness against CD44 receptor-overexpressing breast cancer. A study by Ghaznavi et al. [54] reported the synthesis and characterization of gold@iron oxide photothermal NPs conjugated with folic acid, whose receptors are overexpressed on the surfaces of certain cancer cells. Moreover, NPs can be engineered with cell-penetrating peptides to enhance their internalization.

Organelle-targeting conjugates provide an alternative approach to enhance photothermal therapy. The cell nucleus stands out as a prime target for cancer treatment due to its critical role as the cellular “heart” where genetic information is stored. In the work of Peng et al. [55], a nucleus-targeted PTT strategy was developed by conjugating the transferrin and TAT peptides (TAT: YGRKKRRQRRR) to IONPs. Another attractive cellular target is mitochondria [56,57], which play a pivotal role in various cellular processes.

It should be noted that active targeting correlates with passive targeting, as the receptor–ligand interaction requires them to be within a 0.5 mm distance [58]. Moreover, when interacting with biological materials, NPs can adsorb a protein corona that effectively shields the ligands [59].

Endogenous and exogenous stimuli are also commonly used to achieve targeted delivery and heighten the sensitivity of PTT. Magnetic targeting is one of the most widely investigated stimuli to achieve targeted accumulation in tumor tissue. IONPs can be guided by the magnetic field through the bloodstream and retained at the site of application of the external magnetic field [60]. In a study by Melancon et al. [61], a magnetic field applied externally to the skin led to an approximately two-fold increase in the delivery of SPIO@AuNS NPs into tumors, compared to mice without the magnet. The use of thermal stimuli is also of interest. Wu and colleagues [62] engineered IONPs with thermal-cleavable Azo linkers, facilitating NIR-sensitive doxorubicin (DOX) drug release. Another strategy for targeted tumor therapy is the utilization of endogenous stimuli such as a low pH [63], glutathione [64], metalloproteinases [65], and other inherent characteristics of the tumor environment.

## 5. Strategies to Improve Photothermal Abilities of IONPs

There are various strategies available to boost the photothermal performance of IONPs (Table 2). One approach involves bandgap engineering achieved by doping, which has the potential to significantly amplify the photothermal efficiency. Another method is functionalization with elements that exhibit diverse conversion mechanisms, such as metals or carbon NPs. Furthermore, a commonly employed strategy uses polymer shells with photothermal abilities on IONPs. The NP shell can also be functionalized with photothermal organic dyes.

Incorporating additional elements in semiconductors through doping creates additional energy levels within the band gap, affecting their absorption and emission properties [66]. During bandgap engineering, the emergence of trap-level states that function as centers for charge recombination is possible, which extends the absorption spectrum to longer wavelengths. Another change may be a shift in the position of the valence or conduction band. These changes have the potential to enhance the photoconversion efficiency. In a recent study [67], it was found that Zn_0.4_Fe_2.6_O_4_ exhibited a 22% reduction in the energy gap, with a value of 1.03 eV, compared to Fe_3_O_4_@γ-Fe_2_O_3_, with a value of 1.32 eV, making it a more efficient photothermal agent.

Incorporating materials with distinct conversion mechanisms, such as plasmonic [68] or carbon NPs, represents a promising method to enhance PTT. In their study, Pasciak et al. [69] observed an enhancement in PTT efficacy when Fe_2_O_3_ nanoflowers were decorated with gold NPs. The specific absorption rate (SAR) values showed a 1.8-fold increase (4581 W/g) compared to the application of γ-Fe_2_O_3_ nanoflowers alone (2940 W/g). The plasmonic effect in gold NPs is suggested as an enhancer of heat generation. In another work, Lu et al. [70] demonstrated the efficacy of combining gold nanoflowers with ultrasmall IONPs. They reported significantly improved photothermal conversion efficiency of 82.7% achieved by this composite. Wang et al. [71] developed photothermal–immunotherapeutic NPs by hybridizing Fe_3_O_4_ with reduced graphene oxide. The resultant NPs achieved a tumor surface temperature of 59 °C, outperforming the IONPs alone, which increased the tumor surface temperature to 47 °C.

The utilization of polymer coatings with inherent photoconversion properties benefits PTT. For example, polypyrrole [72] and polydopamine are biocompatible polymers with good absorption in the NIR region. In their work, Wu et al. [73] fabricated superparamagnetic IONPs (SPIONs) with cluster@PDA as a magnetic field-guided cancer theranostic agent. This composite exhibited enhanced photothermal capabilities when compared to SPION clusters without the PDA coating. This improvement could be attributed to the synergistic photothermal conversion capabilities of the IONPs and the PDA in combination. Furthermore, the application of an external magnetic field resulted in heightened cellular uptake, further increasing the efficacy of photothermal therapy.

One of the most used techniques for improved PTT utilizes organic dyes. Such dyes with strong absorption in the optical biological windows can enhance the efficacy of PTT and provide fluorescent imaging. For example, Wang et al. [74] reported the coupling of IR806 dye and Fe_3_O_4_ NPs, which improved the NIR absorption of Fe_3_O_4_ NPs. They found a 3.5-fold increase in the photothermal conversion efficiency of the Fe_3_O_4_-IR806 NPs compared to the Fe_3_O_4_ NPs. An increase in the efficiency of PTT can also be achieved by manipulating the cellular physiology. In their review, Cao et al. [75] describe innovative strategies to enhance PTT at mild temperatures through the regulation of heat shock protein expression, autophagy, and free radical generation.

## 6. IONPs in Theranostics

Theranostics provide the opportunity for both imaging and therapy through precisely engineered nanoagents (Table 3). In the context of IONPs, their magnetic properties lead to their applications as contrast agents in MRI. In addition to enhanced conversion efficiency, the functionalization of NPs enables them to be used as a diagnostic modality. Organic dyes and carbon NPs find utility in fluorescent analysis, while metallic elements can act as contrast agents for computed tomography, photoacoustic tomography (PA), and surface-enhanced Raman scattering (SERS). The integration of multiple imaging techniques holds the potential to significantly enhance the diagnostic outcomes, compensating for the limitations inherent in each technique.

### 6.1. Iron Oxide as PTT and MRI Agent

MRI is one of the main in vivo imaging modalities, with several advantages, such as the use of non-ionizing radiation, high penetration depth, anatomical and functional information, and soft-tissue contrast. Additionally, the image contrast can be further enhanced using contrast agents that increase lesion differentiation from healthy tissue. IONPs, with the benefits of their superparamagnetism and biocompatibility, have already been approved as MRI agents [86,87]. SPIONs, whose hydrodynamic size is greater than 50 nm, are phagocytosed by Kupffer cells in the liver, so they have been used for liver imaging. For example, ferumoxides (Endorem^®^ (Guerbet, Villepinte, France) Feridex^®^ (Advanced Magnetics, Inc., Cambridge, MA, USA) hydrodynamic diameter of 50–100 nm) and ferucarbotran (Resovist^®^ (Bayer Schering Pharma AG, Berlin, Germany) Cliavist^®^ (for the French market) 60–80 nm) have a short blood half-life of 10–12 min and are used for liver tumor and metastasis imaging. Ultrasmall SPIONs, with hydrodynamic diameters smaller than 30 nm, have a longer blood half-life than SPIONs and can target macrophages in the deep compartments. They find application in MR angiography, lymph node imaging, bone marrow imaging, and more. For example, ferumoxtran-10 (Sinerem^®^ (SPL Medical B.V., Nijmegen, The Netherlands) Combidex^®^ (AMAG Pharmaceuticals, Inc., Waltham, MA, USA 30 nm) and ferumoxytol (Feraheme^®^ (AMAG Pharmaceuticals, Inc., Waltham, MA, USA 20–30 nm) have a blood half-time of hours and are used for lymph node metastasis or atherosclerotic plaque imaging, respectively.

The size, the shape, the crystallinity, and the presence of a doping material within the crystal lattices of IONPs play a crucial role in determining the type of contrast (positive or negative) and the magnitude of the magnetic moment [88]. The abundance of magnetite, the increased size, and the shape anisotropy can benefit not only the PTT efficacy but also the MRI capabilities [33]. Maghemite exhibits saturation magnetization of around 80% of that found in magnetite [89]. Like other types of NPs, ligand functionalization provides the ability to selectively target specific tissue types or cells. Lui [90] engineered photothermal theranostic ultrasmall SPIONs (USPIO-PEG-sLex), with MRI imaging abilities, designed to target E-selectin overexpression. This overexpression is linked to tumor progression and the metastasis of a variety of malignant tumors. The results showed a reduction in the T2* relaxation value in the tumor area and confirmed the targeting ability and photothermal conversion of the USPIO-PEG-sLex NPs. Kale et al. [60] described the design of a composite Fe_3_O_4_@GdPB, with an IONP core and gadolinium-containing Prussian blue shell, as a theranostic agent for the T1-weighted MRI and PTT of tumors. Gadolinium is a component of another category of clinical MRI contrast agents. The resultant Fe_3_O_4_@GdPB nanocomplex increased the signal-to-noise ratios in T1-weighted tumor scans and demonstrated the potential to be directed to specific anatomical locations through the external magnetic field. The nanocomposite was found to be an effective PTT agent in an animal model of neuroblastoma.

### 6.2. IONP Composites as PTT and Fluorescence Agents

Fluorescence imaging stands as a promising imaging modality, enabling the visualization of biological structures and processes across various scales, ranging from the molecular and organellar to organ levels. This versatile tool also facilitates the precise localization of single molecules within cells with a nanometric resolution [91]. A diverse range of materials serve as fluorescent elements, including semiconductors like quantum dots, rare earth-doped matrices such as nanophosphors, organic dyes, and various forms of carbon NPs. In the field of medicine, fluorescence imaging serves as a tool for the monitoring of cell migration, the guidance of surgical procedures, the visualization of arterial plaques, the detection of amyloid-β plaques in Alzheimer’s disease, and more.

The integration of photothermia with fluorescence diagnostics has emerged as a prevalent strategy in the field of theranostics. This is attributed to the inherent fluorescent properties of materials like carbon NPs and organic dyes, which also possess photothermal potential. The class of heptamethine dyes, including MHI-148, IR-780, IR-783, and IR-808, has gained significant interest due to their selective accumulation in tumor tissue [92]. Furthermore, some of these dyes can induce mitochondrial toxicity, thereby enhancing the damage caused by photothermia. Chang et al. [93] have developed a dual imaging PTT nanoplatform that combines fluorescence and MRI imaging capabilities. This platform is based on the IR806 dye and IONPs functionalized with citraconic anhydride. The citraconic anhydride acts as a smart pH charge conversion agent that enhances the targeted accumulation in the tumor microenvironment. In addition to the fluorescence provided by IR806, the combination of IONPs and IR806 enhances the light-to-heat conversion efficiency and reduces the required light irradiation dose compared to using IONPs or IR806 alone.

However, there are some limitations when utilizing organic dyes, including their low quantum yield and strong susceptibility to photo-bleaching. These issues have resulted in the exploration of alternative options like quantum dots and carbon NPs. Quantum dots are semiconductor crystals ranging in size from 1 to 10 nm. They exhibit photostability, a narrow emission band, and a good extinction coefficient—more than 10 times larger than those of organic dyes [94]. Combining their fluorescence emission and optical absorbance, these advantages can extend the use of quantum dots as a potential NIR-responsive photothermal agents for imaging-guided PTT. Carbon NPs are also of great interest as theranostic PTT agents due to their excellent mechanical, optical, and thermal properties [95]. Wang et al. [96] constructed magnetic Fe_3_O_4_ NP cores covered by a carbon shell for dual fluorescent/MRI bioimaging and PTT agents. Within the nanocomposite, the Fe_3_O_4_ NPs contributed to contrast enhancement for T2-weighted MRI in vivo, whereas the carbon shell generated confocal fluorescence signals.

### 6.3. IONP Nanocomposites for PTT and SERS

Raman scattering is a well-known technique for molecular identification. Every molecule possesses its own unique Raman spectrum, a characteristic that may give detailed information about its structural and chemical composition. Raman spectroscopy has a low signal-to-noise ratio but it has been found that the Raman signal can be amplified by a factor of 10^6^–10^9^ when the specimen is placed in contact with plasmonic NPs (SERS). Thanks to its remarkable attributes—high sensitivity, precise signal specificity, and resistance against photobleaching—SERS has been widely investigated in the field of disease diagnosis. Furthermore, a combination of MRI and SERS can achieve high sensitivity and provide detailed biological information [97]. The MRI resolution is at the millimeter level and cannot reach the cellular level. On the other hand, SERS has ultra-sensitivity, so dual-mode MRI–SERS can take full advantage of both techniques to improve the diagnostic accuracy. In their study, Huang et al. [76] synthesized photothermal NPs with the capacity for the real-time monitoring of drug release. This nanocomposite provided various functionalities: cancer cell targeting, pH-triggered drug release, MR imaging, SERS-traceable detection, and CT–PTT for cancer treatment. The nanosystem’s architecture involves the deposition of Fe_3_O_4_@Au@Ag NPs onto graphene oxide (GO), coupled with the labelling of a Raman reporter, 4-mercaptophenylboronic acid (4-MPBA). Furthermore, DOX was loaded using a pH-responsive linker, a boronic ester. Within the acidic tumor microenvironment, the pH-sensitive borate ester bond broke, resulting in the release of DOX and real-time changes in the 4-MPBA SERS spectra. Additionally, the strong T2 MRI signal and NIR photothermal efficiency of the NPs make it suitable for MRI and PTT. In another study, Wang et al. [98] used Cu_2_O for SERS sensitivity in a multifunctional Fe_3_O_4_@Cu_2_O theranostics agent for dual MRI-SERS imaging-guided PTT.

### 6.4. IONP Nanocomposites for PTT and Computed Tomography Imaging

Computed tomography imaging, one of the most widely used imaging techniques for tumor diagnosis, is based on differential tissue X-ray attenuation. The larger Z value of iron and its density can ensure the contrast ability of IONPs. Moreover, the potential to enhance the contrast can be increased through NPs’ functionalization with other metals like gold. Li et al. [99] reported HA-targeted Fe_3_O_4_@Au NPs for the tri-mode (MRI/computed tomography/thermal) imaging and PTT of cancer, with targeting abilities for CD44 receptor-overexpressing cancer cells. After the intratumoral administration of the Fe_3_O_4_@Au-HA NPs, the tumor area exhibited a significant increase in brightness 10 min after injection. The computed tomography HU value within the tumor region was measured at 2303.3 HU, a substantial elevation compared to its pre-injection value of 157.4 HU. Wang et al. [100] designed multifunctional theranostic polystyrene@chitosan@gold NP–Fe_3_O_4_ nanocomposites for a spectrum of imaging modalities, including MR, fluorescence, and computed tomography, as well as the application of PTT. Following nanocomposite injection, a bright signal at the tumor site was seen. The increase in the HU computed tomography value was found to correlate with the increased concentration of the PS@CS@Au–Fe_3_O_4_–FA/ICG NPs.

### 6.5. IONP Nanocomposites for PTT and PA Imaging

PA imaging relies on the photoacoustic effect, which occurs when absorbed optical energy is converted into acoustic energy [101]. Acoustic waves exhibit significantly reduced scattering compared to optical waves within tissue; thus, PA can generate high-resolution images. This method also suffers from a relatively limited penetration depth, usually around a few centimeters [64]. Fu et al. [77] designed multifunctional TiS2-based NPs for magnetic targeted dual-modal MRI/PA imaging-guided synergistic PTT–immunotherapy. The nanoplatform could monitor in real time the treatment process via dual PA imaging and T2-weighted MR imaging. In a similar study, Ma et al. [102] developed Fe_3_O_4_–Pd Janus NPs with dual-mode MRI/PA imaging properties for MH–PTT and chemodynamic therapy. It was found that the PA signal intensities of the Fe_3_O_4_–Pd NPs increased linearly with an increase in the Pd concentration.

### 6.6. IONP Nanocomposites for PTT and Ultrasound Imaging

Ultrasound (US) imaging is another widely used diagnostic techniques due to its advantages of being relatively inexpensive, portable, non-invasive, low-cost, and capable of real-time imaging. Li et al. [103] fabricated NPs by loading IONPs into poly(lactic acid) microcapsules, functionalized with graphene oxide. The resulting microcapsules enhanced the contrast in US, MRI, and PA imaging. In similar study, Ke et al. [104] reported multifunctional bimodal US/MRI-guided PTT NPs fabricated by loading perfluorooctylbromide and SPIONs into poly(lactic acid) nanocapsules.

## 7. PTT in Combination with Other Treatment Modalities

The combination of PTT with other therapies can result in synergistic therapeutic effects, significantly enhancing the overall efficacy against tumors. Through hyperthermia, PTT induces tumor cell death via apoptosis, necrosis, and immunogenic cell death (ICD). ICD triggers the release of damage-associated molecular patterns (DAMPs) and tumor-associated antigens (TAAs), stimulating innate and adaptive immune responses. With its precise and targeted approach, PTT minimizes the damage to surrounding healthy tissue. Integrating PTT with other therapies allows a comprehensive cancer treatment strategy with multiple therapeutic benefits (Table 4).

### 7.1. Synergistic CT–PTT

In numerous preclinical studies, the combination of CT and PTT has exhibited synergistic therapeutic benefits with minimal side effects. Hyperthermia acts as a chemosensitizer, improving the drug distribution by enhancing the membrane fluidity and increasing the vascular permeability and blood flow in tumor tissue [105]. Additionally, research has demonstrated that hyperthermia can enhance the cytotoxicity of some CT agents [106]. Therefore, the integration of PTT and CT mediated by NPs represents a promising strategy for tumor theranostics.

Jin et al. [107] used multifunctional IONPs for synergistic CT–PTT. These IONPs were coated with polydopamine, whose abundant functional groups facilitated efficient drug loading alongside its photothermal capabilities. Taking advantage of the porous structure of IONPs, this NP formulation enabled a drug loading capacity exceeding 24.1 wt% and significantly enhanced the antitumor efficacy. In their study, Li et al. [78] synthesized IONPs covalently conjugated with nanodiamonds, with good biocompatibility, photothermal stability, and photothermal conversion efficiency of 37.2%. These NPs demonstrated a high loading capacity for the CT drug DOX, reaching 193 mg/g, and exhibited magnetic navigation as well as pH- and NIR-responsive release characteristics. Their results revealed a synergistic inhibitory effect on tumor cells through combined CT–PTT. Kwon et al. [108] investigated the synergistic anti-cancer effects of Temozolomide- and Indocyanine green-loaded Fe_3_O_4_ NPs by CT–PTT. Temozolomide inhibits the viability of malignant glioma cells while Indocyanine green acts as both a photothermal agent and a photodynamic photosensitizer under NIR laser irradiation. A Western blot analysis and reverse transcription–quantitative polymerase chain reaction demonstrated that these NPs significantly enhanced the anti-cancer effects on U-87 MG glioblastoma cells by modulating intrinsic and extrinsic apoptosis genes.

### 7.2. Synergistic Radiation Therapy–PTT

RT is known to elevate the production of superoxide anions within mitochondria [109], a process that prompts superoxide dismutase to convert them into H_2_O_2_. The presence of IONPs further catalyzes the conversion of H_2_O_2_ into highly reactive hydroxyl radicals, amplifying the generation of ROS within tumor cells and enhancing the efficacy of RT. In addition, the combination of RT and hyperthermia triggers apoptosis through multiple mechanisms, including oxygenation, DNA damage, and cell cycle arrest [110]. The application of heat also enhances the immunological responses against cancer by triggering the release of antigens and cytokines and increasing the activity of immune cells.

In their study, Hu et al. [111] investigated core–shell gold NPs with magnetic targeting abilities for synergistic RT–PTT in cervical cancer. While both RT and PTT inhibited HeLa cell growth, the combined treatment, facilitated by enhanced magnetic delivery, lead to significant damage to most HeLa cells. In another study [112], the researchers reported the synthesis and utilization of three-material inorganic heterostructures composed of iron oxide–Au–copper sulfide. These trimers exhibited multifunctional properties, including optimal heating loss in MH, high adsorption in the first NIR biological window, and serving as carriers for 64Cu at internal RT. Furthermore, they are traceable by positron emission tomography, demonstrating high potential for advanced imaging and therapy.

### 7.3. Synergistic Photodynamic Therapy–PTT

The combination of photodynamic therapy (PDT) and PTT offers a promising approach for the effective treatment of localized tumors [113]. While PDT relies on oxygen-dependent photosensitizers and faces challenges with tumor hypoxia, mild hyperthermia can improve tumor oxygenation, thereby enhancing the subsequent PDT efficacy. Conversely, hypoxia and tumor acidification post-PDT can sensitize cells to PTT. Hyperthermia elevates the mitochondrial ROS levels and reduces the expression of the ATP-binding cassette transporter ABCG2, thus boosting the PDT efficacy by restricting photosensitizer efflux. Additionally, the ROS generated during PDT directly impair heat shock proteins, weakening their protective function against PTT.

In their study, Chiu et al. [114] investigated the therapeutic potential of a nanocomposite as a combined PDT–PTT agent. These nanoagents exhibited high catalytic activity, effectively breaking down H_2_O_2_ into reactive hydroxyl and hydroperoxyl radicals via Fenton-based reactions. Experiments conducted on mice with solid tumors revealed significant therapeutic effects. Coupling NP injection with NIR illumination led to substantial tumor removal without recurrence. The histological examination further confirmed the extensive tumor cell damage following NP application and NIR laser illumination. Yao and Zhou [115] reported chlorin e6 (Ce6)-conjugated IONPs designed for the ablation of glioblastoma cells via combining PDT–PTT. When subjected to 660 nm laser irradiation, the nanoagents produced singlet oxygen, facilitating PDT. Following the incubation of C6 cancer cells with the NPs, a quantitative analysis revealed that the percentages of living cells in the laser- or NP-treated groups were around 99.0%. Conversely, the NP + laser group exhibited a cell death rate of 83.6%, with only 16.4% of cells surviving. In another study, Zhao et al. [79] developed mesoporous Fe_3_O_4_@TiO_2_ microspheres for NIR-light-enhanced chemodynamic therapy, CT, and PDT. Titanium dioxide was utilized as an effective PDT agent. The nanoplatform was further optimized by loading the CT drug DOX, which could be efficiently released upon NIR excitation and slight acidification. In vitro experiments demonstrated the biocompatibility of the nanoplatforms, magnetic targeting, and the synergistic efficacy of this multifaceted cancer treatment.

As mentioned, the efficiency of PDT is limited by the hypoxic tumor microenvironment and low tissue penetration of ultraviolet/visible light. To address these limitations, Zhang et al. [80] combined Fe_3_O_4_@MnO_2_-doped upconversion NPs with black phosphorus nanosheets. The black phosphorus demonstrated strong O_2_ production for PDT when irradiated at 660 nm and high photothermal conversion efficiency when irradiated at 808 nm, enabling them to serve as both PDT and PTT agents. To overcome the lower tissue penetration of 660 nm laser irradiation, black phosphorus was combined with multifunctional upconversion NPs. These NPs can absorb long-wavelength photons and emit shorter-wavelength light to activate photosensitizers. This approach creates a theranostic platform capable of simultaneous PTT/PDT with single 808 nm laser irradiation, self-supporting O_2_ in the tumor microenvironment, and enabling multimodal imaging.

### 7.4. Synergistic Ferroptosis–PTT

Ferroptosis is a regulated form of cell death characterized by the accumulation of ROS, particularly lipid peroxides, leading to oxidative stress-induced cell death. This process is triggered by the depletion of intracellular antioxidant systems or the disruption of lipid metabolism pathways. The iron-dependent nature of ferroptosis is crucial, as iron catalyzes the Fenton reaction, which generates ROS, initiating lipid peroxidation and damaging cell membranes. Combining moderate heat with IONPs could disrupt the redox balance of tumors, increasing the oxidative damage and sensitizing cells to ferroptosis.

Cai et al. [116] investigated pomegranate-like IONPs composed of SPIONs encapsulated within a reduced poly(β-amino ester)–PEG amphiphilic copolymer. This innovative platform facilitates the photothermal conversion and controlled release of iron ions and DOX under mild hyperthermia generated by NIR irradiation. Additionally, iron overload triggers the polarization of macrophages towards an M1 phenotype via the ROS/acetyl-p53 pathway, enhancing the ferroptosis of tumor cells and suppressing tumor growth. M1-type tumor-associated macrophages lead to increased H_2_O_2_ secretion, promoting the peroxidation of polyunsaturated-fatty-acid-containing phospholipids, while also secreting IFNγ, known to induce lipid peroxidation and ferroptosis by inhibiting SLC7A11 expression. Through the synergism of these functions, the authors observed remarkable ferroptosis and growth inhibition in mouse bladder cancer cells.

Yang et al. [81] developed IONPs targeting folic acid and loaded with Dox. These NPs enhanced the ferroptosis efficiency and exhibited highly efficient PTT upon NIR-II irradiation, along with the controlled release of ferric ions and Dox. This treatment resulted in the significant upregulation of cellular respiration and electron transport while downregulating epigenetic pathways, with the overall effect of cellular apoptosis. In vivo validation in 4T1-tumor-bearing Balb/c mice demonstrated that laser irradiation alone did not inhibit tumor growth. In mice treated with NPs but without light irradiation, ferroptosis significantly inhibited tumor growth, similar to basic CT. The most promising treatment outcomes were observed in the group receiving NPs loaded with Dox and combined with 1064 nm laser irradiation, nearly suppressing tumor growth by 98.6%. Notably, even without the CT drug, Fe_2_O_3_ treatments resulted in a high tumor inhibition ratio of 93.8% after laser irradiation. In another study, Cun et al. [117] created a nanosystem that depleted glutathione and amplified •OH production for photo-strengthened peroxidase-like nanocatalytic tumor therapy. Pretreatment with β-Lapachone effectively elevated the H_2_O_2_ levels, which, together with NIR laser treatment, demonstrated significantly enhanced anti-cancer activity. Notably, the in vivo therapy achieved a tumor inhibition rate of 96.4% and complete tumor ablation in two of five animals.

### 7.5. Synergistic MH–PTT

IONP-mediated MH is a novel cancer treatment. In this method, magnetic NPs are applied to generate overheating via Brownian and Neelian relaxations under an alternating magnetic field (AMF) (100–300 kHz). A combination of MH and PTT in a dual thermal treatment could overcome some of the individual limitations to achieve a promising cancer therapy. IONPs have emerged as the principal agent for MH due to their minimal toxicity in cellular systems and multimodal functionality for diagnostic, targeting, and therapeutic purposes.

In their study, Anilkumar et al. [118] developed liposomal NPs containing citric acid-coated IONPs for dual MH–PTT cancer treatment. In vitro cell culture experiments confirmed the efficient passive accumulation of these liposomes in human glioblastoma U87 cells due to their cationic nature. Treatment with dual MH–PTT resulted in a significant reduction in cell viability compared to single-mode NIR or AMF laser treatments. At a maximum concentration of 4.5 mg/mL, individually, the NIR lasers and AMF reduced the cell viability by 32–35%, but, when combined, this effect was enhanced 2.4-fold to 82%. In a similar study, Bao et al. [82] synthesized magnetite vortex NPs coated with polypyrrole for dual-enhanced hyperthermia. The NPs showed excellent hyperthermia effects when simultaneously exposed to the AMF and NIR laser. In animal experiments, the relative tumor volume was notably lower in the group subjected to both NIR and MH, showing significant tumor suppression and nearly complete tumor reduction compared to a single mode of treatment. Lu et al. [119] developed core–shell Fe_3_O_4_@Au magnetic NPs encapsulating cetuximab, a monoclonal antibody targeting EGFR, for targeted magneto-photothermal therapy against glioma cells. In vivo experiments demonstrated significant tumor growth suppression in the dual MH+NIR treatment compared to the single modes. qRT-PCR and Western blot analyses revealed that NP-mediated hyperthermia upregulated the mRNA and protein levels of intrinsic apoptotic markers in glioma cells.

### 7.6. Synergistic Magneto-Mechanical Effect–PTT

The magneto-mechanical effect describes the induction of mechanical motion by particles when subjected to a magnetic field [120]. In cancer treatment, this effect is utilized to apply controlled oscillations of particles that exert a mechanical force on cancer cells to destroy tumors. This therapy offers a highly localized effect, reducing the damage to surrounding healthy cells.

Lopez et al. [121] showed that SPIONs targeting lysosomes with a size of only 6 nm could induce the death of cancer cells. Their study found that the force generated by the NPs was around 3 pN, which was not sufficient for direct membrane disruption, but the mechanical activation of the magnetic NPs induced lysosome membrane permeation, the release of the lysosome content, and cell death.

In their study, Muzzi et al. [122] synthesized star-shaped Au@Fe3O4 NPs with a Au core covered by a magnetite shell for magneto-mechanical stress and photothermia applications. Following the incorporation of these nanostars, the cell viability was notably reduced to 65% after AMF stimulation and to 45% after exposure to light.

In a study by Wu et al. [123], novel hollow magnetic microspheres were synthesized, composed of superparamagnetic Fe_3_O_4_, for multimode cancer therapy. The results demonstrated that the combination of the NPs, magneto-mechanical force, PTT, and PDT under a varying magnetic field and 808 nm laser irradiation led to the elimination of nearly all cancer cells, with approximately 99% cell death.

### 7.7. Synergistic Immunotherapy–PTT

Immunotherapy harnesses adaptive immunity to suppress tumor growth, but the immunosuppressive tumor microenvironment limits its effectiveness. PTT addresses this limitation [124] by directly eliminating tumors, while also enhancing the tumor immunogenicity through ICD, improving the tumor blood flow, promoting immune cell infiltration, and aiding in the distribution of therapeutic molecules within tumors. PTT-induced ICD releases DAMPs and TAAs, stimulating the innate and adaptive immune responses. Furthermore, PTT increases radical formation, while the radicals block heat shock protein production. These mechanisms collectively initiate an inflammatory cascade, facilitating antigen presentation and leading to immune memory against tumor recurrence and metastasis. Tsao et al. [83] developed dual-sensitivity NPs that could combine indoleamine 2,3-dioxygenase (IDO) immunotherapy, nitric oxide (NO) gas therapy, and PTT. S-nitrosoglutathione-loaded gold NPs were coated with IONPs, betanin, and 1-methyl-D-tryptophan. S-nitrosoglutathione was decomposed into NO gas and glutathione disulfide by heat and UV/visible light. NO has several tumoricidal effects and can react with ROS to produce highly reactive nitrogen species. The combination of PTT and NO gas therapy has an excellent cancer therapy effect, resulting in a large amount of TAAs compared to the individual treatment in vitro. These antigens enhance the maturation of dendritic cells, leading to long-term immunity.

In another study, Wang et al. [84] fabricated a tumor microenvironment-responsive nanoplatform for the therapy of aggressive Panc02-H7 pancreatic tumors. In this nanoplatform, IONPs were loaded with an immunoadjuvant (imiquimod, IMQ) and ICG. The PTT induced ICD, which, combined with the released IMQ, triggered antitumor immunity and resulted in reduced metastasis. The nanoplatform induced the maturation of bone marrow-derived dendritic cells, the polarization of macrophages to the M1 phenotype, increased T cell infiltration, and the stimulation of cytokine secretion. The results showed the complete eradication of primary tumors and a prolonged survival time, without damage to normal tissue and systemic autoimmunity.

Zhou et al. [85] developed a nanoplatform containing R837 immunoadjuvant ovalbumin as a strong immunogenic antigen and manganese iron oxide for synergetic PTT–immunotherapy. Under 805 nm laser irradiation, the nanoplatform produced dendritic cell maturation and systemic T-cell activation. The synergistic effect between ovalbumin and R837 stimulated an immune response when combined with the released tumor antigens induced by PTT. These multitasking NPs significantly inhibited lung metastasis and tumor growth. The PTT-treated mice had a survival rate of 33.3% at 100 days after treatment, whereas no mice survived a single treatment.

### 7.8. Synergistic Gene Therapy–PTT

Gene therapy is an innovative approach in cancer treatment, effectively controlling tumor growth by regulating key genes or proteins [125]. However, the direct administration of gene drugs without a delivery vehicle makes them susceptible to degradation by nucleases. Moreover, the lack of tumor-targeting abilities of naked genes may result in unintended effects on healthy cells and tissue. The challenges posed by the negative charges, molecular weight, and hydrophilicity of naked genes further hinder their penetration into cancer cells. Therefore, the selection of an appropriate gene delivery system is crucial for the success of cancer therapy [126]. IONPs offer the potential for efficient delivery to tumors due to their biocompatibility, surface modification capabilities, and magnetic responsiveness. IONPs, when surface-modified, can effectively load negatively charged genetic drugs through electrostatic adsorption and have the potential to enhance the tumor treatment by combining gene therapy with phototherapy. Laser irradiation can improve gene delivery across membranes, a novel approach known as photoporation [127], via short pulses that facilitate the entry of NPs loaded with siRNA into cells. The temperature elevation caused by the conversion of light into thermal energy triggers the formation of small water vapor bubbles, which expand, collapse, and generate high-pressure shockwaves. These shockwaves create pores in nearby cell membranes, allowing the NPs to enter the cells through diffusion.

Huang et al. [128] applied the combination of porous IONP-mediated PTT and gene therapy to destroy lung cancer cells in vitro and in vivo. Long noncoding RNA CRYBG3, induced by heavy ion irradiation in lung cancer cells, has been shown to destroy the actin cytoskeleton, decrease invasiveness, induce cell death, and inhibit tumor progression. Combining PTT with CRYBG3-mediated gene therapy resulted in synergistic cancer cell-killing efficacy, outperforming either therapy alone.

## 8. Conclusions and Future Directions

IONPs possess a set of characteristics that make them valuable for various medical applications. Their magnetic properties enable them to serve as contrast agents for MRI, mediators for MH, and delivery vehicles through magnetoporation and magnetic navigation. In addition, they exhibit responsiveness to various physical fields. When exposed to laser irradiation, IONPs efficiently convert light into heat, inducing localized hyperthermia. Their catalytic nature enhances ROS production, leading to oxidative stress and ferroptosis.

The potential of IONPs in medical applications appears promising, with several key areas for further research and development. IONP research on magnetic navigation holds promise in improving the precision and efficiency of drug delivery systems. Such advances could greatly increase the effectiveness of treatment while minimizing adverse side effects. Expanding the use of IONPs as multimodal contrast agents is an important direction of development. Each imaging technique has unique advantages and limitations, making the use of a combination of modalities to obtain a comprehensive picture of the disease common practice. The ability of IONPs to enhance various imaging modalities can be exploited to provide more comprehensive diagnostic information, improving the accuracy of disease detection.

The synergistic effects of combining IONPs with other therapies, such as RT, PDT, and CT, also offer the possibility to increase the efficacy of cancer treatment. Understanding how IONPs modulate the immune response opens new avenues in cancer immunotherapy. The enhancement of ICD induced by IONPs has the potential to lead to stronger and long-lasting antitumor immunity. This can significantly affect the treatment of metastatic tumors and improve patients’ prognosis. The multifunctionality of IONPs positions them as useful mediators in numerous medical applications, offering flexibility and potential for healthcare innovation.

## Figures and Tables

**Table 1:** Some examples of methods for IONP synthesis.

Method	Nanoparticle	Size	Shape	Cell Line	Application	Ref.
Laser ablation	Fe_3_O_4_	15 nm	spherical, hexagonal	-	-	[16]
Galvanic reaction, etching	Copper–IONPs	14 nm	porous, hollow	4T1 cells L02 cells	chemodynamic, PTT, and chemotherapy	[17]
Flame spray pyrolysis	Carbon-encapsulated Au–Fe_3_O_4_	51.9–55.2 nm	spherical	esophageal cancer cells	MH and PTT	[18]
Single-pass aerosol route	Au–Fe_3_O_4_	24.7 nm	square-like	HEK 293 cells	CT—MRI dual-mode imaging, gene delivery, and PTT	[19]
Coprecipitation	Fe_3_O_4_-loaded alginate hydrogel	61.3 nm	-	CT26 cells	PTT	[20]
Hydrothermal method	Ag@Fe_3_O_4_	250 nm	spherical	SKOV3	PTT	[21]
Microwave-assisted polyol method	γ-Fe_2_O_3_	44–162 nm	nanoflowers	-	MH and PTT	[22]
Green synthesis	Au–IONP composite	19 nm	spherical	HeLa cells	MH and PTT	[23]

**Table 2:** Advantages and applications of different strategies to improve the photothermal abilities of IONPs.

Type of Modification	Mechanism of Action	Application in Cancer Therapy
Bandgap engineering	Narrowing of bandgap through doping-induced formation of energy states	Enhanced photothermal performance of NPs
Incorporation of elements that exhibit diverse conversion mechanisms	Combination of independent photothermal conversion mechanisms in hybrid NPs	Optimized absorption spectrum Multimodal imaging
Polymer coating on NPs with photothermal ability	Additional layer for light absorption and heat generation	Enhanced photothermal conversion efficiency; improved stability due to polymer shell, targeting, immune escape, drug loading, etc.
Photothermal organic dyes on NPs	Combination of two photothermal agents/photodynamic and photothermal agents	Optimized absorption spectrum; PTT–PDT therapy; fluorescence imaging

**Table 3:** Some examples of multifunctional IONPs in cancer therapy.

Nanocomposite	Functionalization	Responsiveness	Diagnostic Mode	Therapy Mode	Ref.
Fe_3_O_4_–GO	EGFR	magnetic targeting	-	chemo-PTT	[52]
MnFe_2_O_4_	macrophage cell membrane, DOX		-	chemo-PTT	[63]
PMP@USPIO	MMP9-sensitive peptides, DOX	MMP-9	MRI	chemo-PTT	[65]
Au–USPIO	-	-	MRI/CT/ PA	PTT–RT	[70]
Fe_3_O_4_–rGO	-	-	MRI	PTT–immunotherapy	[71]
Fe_3_O_4_	IR806	-	-	PTT–PDT	[74]
Fe_3_O_4_@Au@ Ag–GO	boronic ester DOX	pH-responsive	SERS/MRI	chemo-PTT	[76]
TiS_2_–IONPs	-	magnetic targeting	PA/MRI	PTT–immunotherapy	[77]
Fe_3_O_4_	nanodiamonds DOX	pH and NIR-responsive	MRI	chemo-PTT	[78]
Fe_3_O_4_@TiO_2_	DOX	magnetic targeting pH and NIR-responsive	-	CDT/PDT/PTT/ chemotherapy	[79]
Fe_3_O_4_@MnO_2_	chlorin e6 black phosphorus	-	MRI/ fluorescence/PA/US	PTT–PDT	[80]
USPIO Fe_2_O_3_	folic acid DOX	pH-responsive	MRI	PTT–chemotherapy–ferroptosis	[81]
Fe_3_O_4_@PPy–PEG	PPy	-	MRI/PA	MH–PTT	[82]
AuNPs–Fe_3_O_4_	GSNO/betanin 1-M-DT	pH-responsive	-	NO gas–PTT–immunotherapy	[83]
IMQ@IONs/ICG	ICG/ imiquimod	-	MRI	PTT–immunotherapy	[84]
MnFe_2_O_4_	R837 ovalbumin	-	MRI	PTT–immunotherapy	[85]

**Table 4:** Comparison of common combinations of PTT with other treatment modalities.

Synergistic Therapy	Key Features	Mechanism of Action	Advantages
CT–PTT	Hyperthermia acts as a chemosensitizer, making chemotherapy more effective	Improves drug distribution by enhancing membrane fluidity and increasing vascular permeability	Better targeting of tumors, minimizing the damage to surrounding healthy tissue
Radiation Therapy–PTT	Reduced radiation resistance Potentially reduced radiation dose	IONPs amplify the generation of ROS within tumor cells, enhancing the efficacy of RT; hyperthermia increases blood flow and oxygenation	Enhances the effects of radiation, leading to more effective tumor control
Photodynamic Therapy–PTT	Improves the overall effectiveness of both therapies	Mild hyperthermia improves tumor oxygenation, enhancing PDT efficacy; ROS generated during PDT impair heat shock proteins, weakening their protective function against PTT	This synergy may result in fewer systemic side effects and lower doses than either therapy used alone
MH–PTT	The magnetic field penetrates deeper into the tissue compared to laser irradiation	Ensures more comprehensive tumor treatment by effectively targeting both surface and deeper tumor regions	Provides a dual thermal treatment that overcomes individual deficiencies
Immunotherapy–PTT	PTT enhances tumor immunogenicity	Iron overload triggers the polarization of macrophages towards an M1 phenotype	Enhances immune memory against tumor recurrence and metastasis
